# Effects of Strength Training Using Unstable Surfaces on Strength, Power and Balance Performance Across the Lifespan: A Systematic Review and Meta-analysis

**DOI:** 10.1007/s40279-015-0384-x

**Published:** 2015-09-10

**Authors:** David G. Behm, Thomas Muehlbauer, Armin Kibele, Urs Granacher

**Affiliations:** School of Human Kinetics and Recreation, Memorial University of Newfoundland, St. John’s, NL Canada; Division of Training and Movement Sciences, Research Focus Cognition Sciences, University of Potsdam, Am Neuen Palais 10, Building 12, 14469 Potsdam, Germany; Institute for Sports and Sport Science, University of Kassel, Kassel, Germany

## Abstract

**Background:**

The effectiveness of strength training on unstable surfaces (STU) versus stable surfaces (STS) or a control condition (CON; i.e. no training or regular training only) for strength, power and balance performance across the lifespan has not yet been investigated in a systematic review and meta-analysis.

**Objective:**

The aims of this systematic review and meta-analysis were to determine the general effects of STU versus STS or CON on muscle strength, power and balance in healthy individuals across the lifespan and to investigate whether performance changes following STU are age specific.

**Data Sources:**

A computerized systematic literature search was performed in the electronic databases PubMed and Web of Science from January 1984 up to February 2015.

**Study Eligibility Criteria:**

Initially, 209 articles were identified for review. Only controlled trials were included if they investigated STU in healthy individuals and tested at least one measure of maximal strength, strength endurance, muscle power, or static/dynamic balance. In total, 22 studies met the inclusion criteria.

**Study Appraisal and Synthesis Methods:**

The included studies were coded for the following criteria: age, sex, training status, training modality, exercise and test modality. Effect size measures included within-subject standardized mean differences (SMD_w_) and weighted between-subject standardized mean differences (SMD_b_). Heterogeneity between studies was assessed using *I*^2^ and *χ*^2^ statistics. The methodological quality of each study was assessed using the Physiotherapy Evidence Database (PEDro) Scale.

**Results:**

Our search failed to identify studies that examined the effects of STU versus STS or CON in children and middle-aged adults. However, four studies were identified that investigated the effects of STU versus CON or STS in adolescents, 15 studies were identified in young adults and three studies were identified in old adults. Compared with CON, STU produced medium effects on maximal strength in young adults and no effects to medium effects in old adults. In addition, large effects were detected on strength endurance in adolescents and in young adults; in old adults, a small effect was found. With regard to muscle power, medium effects were observed in young adults and small effects were observed in old adults. Further, large effects were found for static and dynamic balance in old adults, but only a small effect was found for dynamic balance in young adults. The comparison of STU and STS revealed inconsistent results as indicated by training-induced changes in favour of STU, as well as STS. Small to medium effects were found for maximal strength in adolescents in favour of STS, and small effects were found in young adults in favour of STU. With regard to strength endurance, large effects were found in adolescents in favour of STS and small effects were found in favour of STU. Additionally, we detected small effects in young adults in favour of STU. In terms of muscle power, no effects were observed in adolescents but medium effects were found in favour of STS in young adults. With regard to balance, small effects were detected in adolescents for static and dynamic balance in favour of STU. In young adults, small effects were found for static balance in favour of STS. With regard to dynamic balance, the analysis revealed small effects in young adults in favour of STU.

**Limitations:**

The quality of the included studies was rather low, with mean PEDro scores of 5.8, 4.0 and 5.0 for studies including adolescents, young adults and old adults, respectively. Further, trivial to considerable heterogeneity between studies (i.e. 0 % ≤ *I*^2^ ≤ 96 %) was detected.

**Conclusions:**

Compared with CON, STU is effective in improving muscle strength, power and balance in adolescents, young adults and old adults. However, inconsistent results were particularly found in adolescents and young adults when the specific effects of STU were compared with those of STS. We conclude that the performance of STU compared with STS has limited extra effects on muscle strength, power and balance performance in healthy adolescents and young adults. Given that our systematic search did not identify studies that examined the effects of STU versus STS in children, middle-aged adults and old adults, further research of high methodological quality is needed to determine whether there are additive effects of STU as compared with STS in those age groups.

## Key Points

**Table:** 

This systematic review and meta-analysis determined the effects of strength training on unstable surfaces (STU) versus stable surfaces (STS) or a control condition (CON) on measures of muscle strength, power and balance, administered in the form of controlled trials in healthy individuals across the lifespan (aged ≥6 years).
Our analyses revealed that STU, as compared with CON, is an effective means to improve measures of muscle strength in healthy adolescents, young adults and old adults, and to improve variables of power and balance in young and old adults.
In adolescents and young adults, the specific comparison of STU with STS resulted in contradictory findings, and thus the use of unstable as compared with stable surfaces during strength training is not recommended in healthy adolescents and young adults if the goal is to enhance performance on stable surfaces.

## Introduction

Devices with varying degrees of instability are frequently employed for athletic and everyday performance enhancement, balance promotion and musculoskeletal health to mimic the demands of the various tasks in applied settings [1, 2]. There are many devices that attempt to provide an unstable surface. These devices include air-pressurized balls (e.g. Swiss, physio or exercise balls), hemispherical balls with an inflated dome side and a hard rubber flat side (e.g. the BOSU^®^ ball), inflatable discs, wobble or balance boards, foam tubes, and high- and low-density foam platforms, as well as many other related devices. Unstable devices promote postural disequilibrium or imbalance, as postural sway may project the centre of mass beyond the device’s area of support. Unstable devices also promote postural disequilibrium as the surface distorts (e.g. a low-density foam cushion) readily in response to the reaction forces associated with changes in the centre of pressure.

According to the principle of training specificity [3, 4], training must simulate as closely as possible the demands of the task or activity. Tasks such as sports and fitness activities, occupational tasks and activities of daily living often occur on relatively unstable surfaces (e.g. skiing, snowboarding, skating, walking and working in icy or muddy conditions). Willardson [5] stated that “the optimal method to promote increases in balance, proprioception and spinal stability for any given sport is to practice the skill itself on the same surface on which the skill is performed in competition”. Similarly, Schmidtbleicher [6] stated that intermuscular coordination can only be developed by practising the movement for which coordination is sought. Unfortunately, with some seasonal sports, specific training is not possible year round (e.g. skiing in the summer or baseball in the snow). Therefore, alternative challenges using unstable surfaces could be included in training to provide a progressive overload and to stimulate strength and balance adaptations. Strength training on stable surfaces (STS), such as squats, deadlifts and Olympic lifts, is conducted with a moderate degree of instability [1, 7–10]. Greater degrees of instability are provided when strength training is conducted on unstable surfaces (STU) or with unstable implements.

Proponents of unstable devices suggest that the greater instability may stress the neuromuscular system to a greater extent than STS [11, 12]. The rationale is that destabilizing training environments may enhance neuromuscular adaptations and training specificity, while providing a more varied and effective training stimulus. The Canadian Society for Exercise Physiology position stand [7] indicates that there are functional health benefits of STU (e.g. improved joint stability and reduced lower-extremity injury rates). In addition, improved strength, balance and functional performance have been reported following STU in primarily young healthy adults [13]. Further, STU appears to be a suitable training regimen to be implemented in the rehabilitative context and/or the geriatric context [8, 9]. In fact, the application of STU is not restricted to young healthy adults. For example, more ‘vulnerable’ cohorts due to biological aging (i.e. seniors) or maturational processes (i.e. children, adolescents) may particularly benefit from STU because surface instability allows and demands lower training loads but at the same time sufficiently and adequately stimulates the neuromuscular system of youth and seniors [1, 2, 7–9]. Given that only a few studies have investigated the effects of STU as a promising training regimen in seniors, more research is needed to elucidate the effects of STU in seniors and to find out whether it is more effective than traditional STS. Further, the biological concept of ‘critical or sensitive maturational periods’, i.e. periods during which ontogenetic development reaches a qualitatively new level that provides opportunities for further improvement of an organ, tissue and/or physiological function [14], may imply that the adaptive potential following STU is also high in youth. In fact, Behm and Colado Sanchez [2] propagated the use of STU for performance enhancements in youth. However, it is not known whether increases in muscle strength, power and balance performances are comparable across the lifespan. A lifespan approach appears to be important because experts have reported in narrative reviews that STU is a meaningful and promising training regimen for youth, adults and seniors [2, 15]. However, these statements lack verification, which is why there is a need for this meta-analysis across the age continuum. Further, meta-analyses represent the highest evidence level on the evidence pyramid [16]. Given that this topic has been addressed by narrative reviews only [2, 15], a meta-analysis may further our knowledge in this area by providing in-depth information (representing a high level of evidence).

Therefore, a synthesis of the literature is needed to determine whether or not STU provides additional effects on measures of muscle strength, power and balance in comparison with STS. The purpose of the present systematic review and meta-analysis was to provide a study on the highest evidence level in evidence-based medicine regarding the effects of STU on measures of muscle strength, power and balance administered in the form of controlled trials in healthy individuals across the lifespan. It is hypothesized that STU produces similar or even larger performance enhancements than STS because the performance of STU is highly demanding for the neuromuscular system (i.e. additional joint and postural stability are needed during exercise). Further, on the basis of expert opinion [2, 15] and selected studies [17, 18], we expected that STU would be particularly suitable and effective in seniors and youth because it has previously been shown that strength training using low loads produced similar or even larger performance gains in these age groups.

## Methods

We followed the Preferred Reporting Items for Systematic Reviews and Meta-Analysis (PRISMA) statement guidelines provided by Moher et al. [19] when conducting our systematic review and meta-analysis.

### Literature Search

We performed a computerized systematic literature search in PubMed and Web of Knowledge from January 1984 up to February 2015 to capture all relevant articles that investigated the effectiveness of STU versus STS. The following Boolean search strategy was applied using the operators ‘AND’, ‘OR’ and ‘NOT’: ((‘instability resistance training’ OR ‘instability strength training’ OR ‘free-weight training’ OR ‘instability weight-bearing exercise program’ OR ‘instability weight-bearing strengthening program’ OR ‘instability weight-lifting exercise program’ OR ‘weight-lifting strengthening program’) AND (balance OR ‘balance performance’ OR posture OR ‘postural balance’) AND (‘strength’ OR ‘muscle strength’ OR ‘muscular strength’ OR power OR ‘muscle power’ OR ‘muscular power’) NOT (‘natural surfaces’ OR ‘unilateral exercises’)). The search was limited to the English language, the human species and full-text availability of original articles reporting a controlled trial in an academic journal. Further, we checked the reference lists of each included article, and we analysed relevant review articles [1, 2, 5, 7–9, 20, 21] in an effort to identify additional suitable studies for inclusion in the database.

### Selection Criteria

To be eligible for inclusion, studies had to meet the following criteria: (1) the participants in the experimental groups had to be healthy subjects; (2) the participants had to be aged ≥6 years; and (3) at least one strength, power and/or balance performance outcome had to be reported in the study. Studies were excluded if (1) they did not have a controlled study design; (2) they included patients or people with diseases; or (3) it was not possible to extract means and standard deviations from the results section (i.e. text, tables or graphs) or the authors did not respond to our inquiries. On the basis of the defined inclusion and exclusion criteria, two independent reviewers (TM, UG) screened potentially relevant papers by analysing the titles, abstracts and full texts of the respective articles to elucidate their eligibility.

### Coding of Studies

Each study was coded for the following variables: number of participants, sex, age, training status (i.e. trained or untrained subjects), type of sport practised and experimental groups [i.e. STU, STS or a control condition (CON; i.e. no training or regular training only)]. We coded interventions according to the applied training modalities, i.e. the training period (number of training weeks), training frequency (number of training sessions/week), training volume (number of sets/repetitions/duration per exercise, duration of a single training session) and training intensity [e.g. percentage of one-repetition maximum (1RM)]. If exercise progression was realized over the training period, the range of sets, repetitions or durations per exercise/session was stated. If training modalities were not reported in detail, the authors were contacted and missing information was requested. Our analyses focused on muscle strength, power and balance outcomes. Muscle strength findings were considered in terms of the following categories: maximal strength (e.g. 1RM), strength endurance (e.g. number of sit-ups) and power (e.g. jump height). Balance was classified as either static (e.g. time during a one-legged stance) or dynamic (e.g. timed walking distance). For studies that reported multiple parameters within one of these outcome categories, the most representative parameter was included in our analysis. In terms of muscle strength, 1RM was defined as the most important variable representing maximal strength. With regard to strength endurance, the number of sit-ups was used, and for muscle power, the countermovement jump (CMJ) height was applied. Concerning balance, the time during a one-legged stance was used as a proxy for static balance and the timed walking distance was used as a measure of dynamic balance. If the included studies did not report the results (i.e. means and standard deviations) of pre- and post-testing, we contacted the authors of those studies. In three cases [13, 22, 23], the authors responded and provided the relevant data. If the authors did not respond, the respective studies [24, 25] were excluded.

### Assessment of Methodological Quality and Statistical Analyses

The methodological quality of all eligible intervention studies was assessed using the Physiotherapy Evidence Database (PEDro) Scale. The PEDro Scale rates internal study validity and the presence of statistical replicable information on a scale from 0 to 10, with ≥6 representing a cut-off score for high-quality studies [26]. The predetermined cut-off score of ≥6 points was not a criterion for studies to be included or excluded. Two independent reviewers (UG, TM) performed quality assessments of the included studies. When any disagreement between the raters occurred, a consensus meeting was held and an additional rating was obtained from a third assessor (DGB) to achieve a consensus.

To verify the effectiveness of STU and STS for measures of muscle strength, power and balance, we computed within-subject standardized mean differences as SMD_w_ = (mean pre-test value − mean post-test value)/standard deviation pre-test value, and between-subject standardized mean differences as SMD_b_ = (mean post-test value in intervention group − mean post-test value in control group)/pooled variance [27]. In addition, the standardized mean difference was adjusted for the respective sample size according to the following formula: $g = \left( {1 - \frac{3}{{4N_{i} - 9}}} \right)$, with *N*_*i*_ representing the total sample size [27, 28]. Furthermore, the included studies were weighted with respect to the magnitude of the respective standard error, using Review Manager version 5.3.4 (The Nordic Cochrane Centre, The Cochrane Collaboration; Copenhagen; 2008). A random-effects meta-analysis model was applied to compute the overall standardized mean difference in Review Manager version 5.3.4. As a function of the respective outcome measure (e.g. 1RM, timed walking distance), SMD_w_ and SMD_b_ could be negative or positive. Thus, a positive SMD_w_ value would indicate performance improvements (i.e. an increase in jump height) from pre- to post-intervention within one experimental group, and a negative SMD_w_ value would indicate a performance decrement (i.e. a decrease in jump height) within one experimental group. In terms of SMD_b_, a positive value would be indicative of a performance change in favour of STU, whereas a negative value would be indicative of a performance change in favour of STS or CON. According to Cohen [29], values for SMD_w_/SMD_b_ of 0.00 ≤ 0.49 indicate small effects, values of 0.50 ≤ 0.79 medium effects and values of ≥0.80 large effects. Heterogeneity between studies was assessed using *I*^2^ and *χ*^2^ statistics. On the basis of the recommendations from Deeks et al. [30], values of 0 % ≤ *I*^2^ ≤ 40 % would indicate trivial heterogeneity, values of 30 % ≤ *I*^2^ ≤ 60 % moderate heterogeneity, values of 50 % ≤ *I*^2^ ≤ 90 % substantial heterogeneity and values of 75 % ≤ *I*^2^ ≤ 100 % considerable heterogeneity.

## Results

### Study Characteristics

**Fig. 1:**
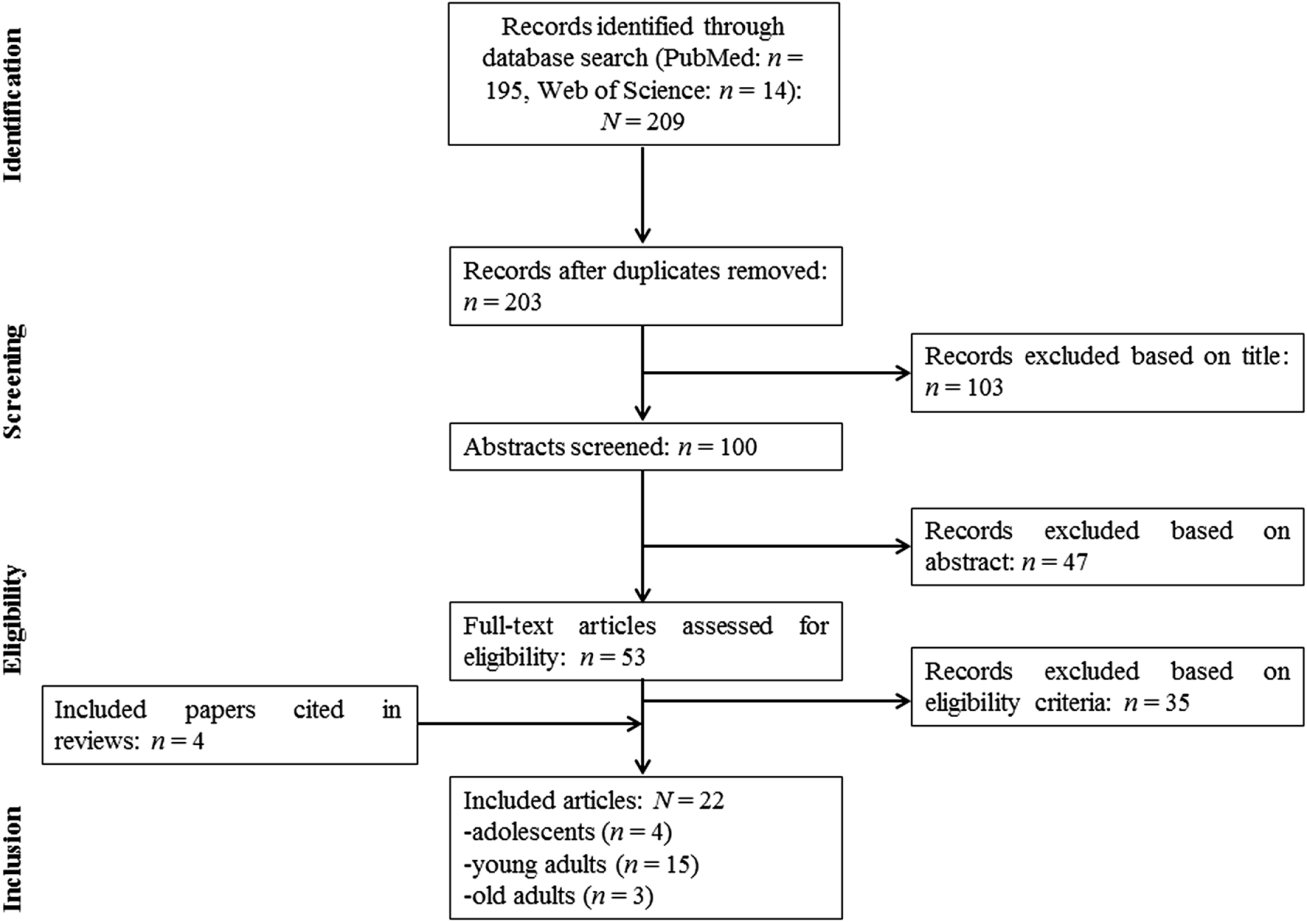
Flowchart illustrating the different phases of the search and study selection

Figure 1 displays a flow chart summarizing our systematic search, which identified a total of 209 controlled trials. After removal of duplicates and exclusion of ineligible articles, 18 studies remained. We identified four additional articles [31–34] from the reference lists of the included papers and from already published review articles [1, 2, 5, 7–9, 20, 21]. Therefore, 22 studies were included in the final analysis. Our lifespan approach was limited to adolescents (four studies), young adults (15 studies) and old adults (three studies) because our systematic search did not reveal any studies investigating the effects of STU on muscle strength, power and balance performance in children and middle-aged adults. Table 1 displays the main characteristics of the 22 included studies.

**Table 1:** Studies examining the effects of strength training on unstable surfaces on measures of muscle strength, power and balance in healthy individuals

References	No. of subjects and sex; age; training status; sport	Groups/training devices	Training modality: no. of training weeks/sessions; no. of sets/reps/duration per exercise; single session duration; training intensity	Exercises	Test modality	Results
Adolescents						
Stanton et al. [35]	18 M; 16 ± 1 years; trained; basketball and football	STU (*n* = 8): normal physical training + core strength exercises on unstable surface (i.e. Swiss ball) CON (*n* = 10): normal physical training only	6 weeks/12 sessions; 2–3 sets of 8–10 reps; 25 min; N/A	Lunge, supine lateral roll, supine leg bridge, superman, forward roll on knees, supine Russian twist	Prone stabilization core stability test	STU-pp: 41.1 % (SMD_w_ 2.08) CON-pp: −6.7 % (SMD_w_ −0.28) STU-CON: SMD_b_ 3.86 (95 % CI 2.16, 5.57)
Granacher et al. [37]	27 (13 M, 14 F); 14 ± 5 years; untrained	STU (*n* = 14): core strength exercises on unstable surface (i.e. Swiss ball, Dynair^®^ cushion) STS (*n* = 13): core strength exercises on stable surface	6 weeks/12 sessions; 3 sets of 20–25 reps or 40–50 s; 30 min; N/A	Curl-up, side bridge, quadruped	Bourban trunk muscle strength endurance test (ventral, dorsal, lateral left/right side)	Ventral: STU-pp: 22.4 % (SMD_w_ 0.45) STS-pp: 14.0 % (SMD_w_ 0.25) STU-STS: SMD_b_ 0.24 (95 % CI −0.52, 1.00) Dorsal: STU-pp: 33.4 % (SMD_w_ 0.79) STS-pp: 41.1 % (SMD_w_ 0.64) STU-STS: SMD_b_ −1.49 (95 % CI −2.35, −0.62) Lateral left side: STU-pp: 8.0 % (SMD_w_ 0.37) STS-pp: 10.5 % (SMD_w_ 0.24) STU-STS: SMD_b_ 0 (95 % CI −0.75, 0.75) Lateral right side: STU-pp: 7.9 % (SMD_w_ 0.31) STS-pp: 9.0 % (SMD_w_ 0.22) STU-STS: SMD_b_ −0.04 (95 % CI −0.80, 0.71)
					SLJ	STU-pp: 3.0 % (SMD_w_ 0.30) STS-pp: 1.1 % (SMD_w_ 0.04) STU-STS: SMD_b_ 0.56 (95 % CI −0.21, 1.33)
					1-legged stance with eyes closed and on foam ground (left/right leg)	Left leg: STU-pp: 20.2 % (SMD_w_ 0.39) STS-pp: 1.9 % (SMD_w_ 0.03) STU-STS: SMD_b_ 0.03 (95 % CI −0.73, 0.78) Right leg: STU-pp: −11.5 % (SMD_w_ −0.17) STS-pp: −12.2 % (SMD_w_ −0.23) STU-STS: SMD_b_ 0.27 (95 % CI −0.49, 1.03)
					YBT (left/right leg)	Left leg: STU-pp: 1.7 % (SMD_w_ 0.19) STS-pp: 2.7 % (SMD_w_ 0.26) STU-STS: SMD_b_ 0.11 (95 % CI −0.65, 0.86) Right leg: STU-pp: 1.9 % (SMD_w_ 0.22) STS-pp: 2.8 % (SMD_w_ 0.28) STU-STS: SMD_b_ 0.12 (95 % CI −0.64, 0.87)
Prieske et al. [36]	37 M; 17 ± 1 years; trained; soccer	STU (*n* = 18): soccer training + core strength exercises on unstable surface (i.e. Swiss ball, Dynair^®^ cushion) STS (*n* = 19): soccer training + core strength exercises on stable surface	9 weeks/18–27 sessions; 2–3 sets of 15–20 reps or 15–20 s; 30 min; N/A	Prone plank, crunches, shoulder bridge, back extension, side bridge	MIMS trunk flexors/extensors	Trunk flexors: STU-pp: −1.1 % (SMD_w_ −0.07) STS-pp: 3.7 % (SMD_w_ 0.27) STU-STS: SMD_b_ −0.66 (95 % CI −1.33, 0) Trunk extensors: STU-pp: 3.8 % (SMD_w_ 0.34) STS-pp: 6.8 % (SMD_w_ 0.41) STU-STS: SMD_b_ −0.28 (95 % CI −0.93, 0.37)
					CMJ	STU-pp: 0.9 % (SMD_w_ 0.09) STS-pp: −1.4 % (SMD_w_ −0.15) STU-STS: SMD_b_ −0.40 (95 % CI −1.05, 0.26)
Granacher et al. [38]	24 M; 14 ± 5 years; trained; soccer	STU (*n* = 12): soccer training + plyometric exercises on unstable surface (e.g. balance pad) STS (*n* = 12): soccer training + plyometric exercises on stable surface	8 weeks/16 sessions; 3–5 sets of 5–8 reps; 30–35 min; N/A	Bilateral CMJ, DJ; bilateral hurdle CMJ, DJ	CMJ	STU-pp: 4.5 % (SMD_w_ 0.45) STS-pp: 12.9 % (SMD_w_ 1.11) STU-STS: SMD_b_ −0.07 (95 % CI −0.87, 0.73)
					DJ	STU-pp: 7.9 % (SMD_w_ 0.59) STS-pp: 11.1 % (SMD_w_ 0.72) STU-STS: SMD_b_ 0.31 (95 % CI −0.50, 1.12)
					Multiple 5 Bounds test	STU-pp: 3.8 % (SMD_w_ 0.43) STS-pp: 3.4 % (SMD_w_ 0.55) STU-STS: SMD_b_ −0.08 (95 % CI −0.89, 0.72)
					1-legged stance with eyes opened and on firm ground (dominant leg)	STU-pp: 14.8 % (SMD_w_ 0.54) STS-pp: 6.7 % (SMD_w_ 0.23) STU-STS: SMD_b_ 0.15 (95 % CI −0.65, 0.95)
					SEBT (composite score for left/right leg)	Left leg: STU-pp: 5.1 % (SMD_w_ 0.58) STS-pp: 6.7 % (SMD_w_ 1.29) STU-STS: SMD_b_ −0.19 (95 % CI −1.00, 0.61) Right leg: STU-pp: 5.5 % (SMD_w_ 0.64) STS-pp: 6.6 % (SMD_w_ 1.13) STU-STS: SMD_b_ 0.03 (95 % CI −0.77, 0.83)
					1-legged perturbed stance with eyes opened and on firm ground (dominant leg)	Mediolateral oscillations: STU-pp: 33.3 % (SMD_w_ 0.76) STS-pp: 26.6 % (SMD_w_ 0.81) STU-STS: SMD_b_ 0.01 (95 % CI −0.79, 0.81) Anterior–posterior oscillations: STU-pp: 56.2 % (SMD_w_ 0.81) STS-pp: 35.1 % (SMD_w_ 0.72) STU-STS: SMD_b_ 0.21 (95 % CI −0.59, 1.01)
Young adults						
Stanforth et al. [31]	55 F; 20–40 years; untrained	STU (*n* = 20): core strength exercises on unstable surface (i.e. Resist-A-Ball^®^) STS (*n* = 15): core strength exercises on stable surface CON (*n* = 20): no intervention	10 weeks/20 sessions; 2 sets of 10–50 reps; N/A; N/A	Crunches, oblique twists, back extension	Double leg-lowering test	STU-pp: 49.6 % (SMD_w_ 1.29) STS-pp: −13.5 % (SMD_w_ −0.34) CON-pp: −13.7 % (SMD_w_ −0.51) STU-STS: SMD_b_ 1.30 (95 % CI 0.56, 2.04) STU-CON: SMD_b_ 1.24 (95 % CI 0.56, 1.93)
					Trunk flexion/extension	Trunk flexion: STU-pp: 94.8 % (SMD_w_ 1.66) STS-pp: 113.8 % (SMD_w_ 1.83) CON-pp: 75.6 % (SMD_w_ 1.55) STU-STS: SMD_b_ 0.29 (95 % CI −0.38, 0.97) STU-CON: SMD_b_ 0.95 (95 % CI 0.29, 1.60) Trunk extension: STU-pp: 156.2 % (SMD_w_ 3.17) STS-pp: 120.6 % (SMD_w_ 2.56) CON-pp: 66.7 % (SMD_w_ 1.31) STU-STS: SMD_b_ 0.46 (95 % CI −0.22, 1.13) STU-CON: SMD_b_ 1.40 (95 % CI 0.70, 2.10)
Carter et al. [42]	20 (sex N/A); 38 ± 9 years; untrained	STU (*n* = 10): core strength exercises on unstable surface (i.e. Resist-A-Ball^®^) CON (*n* = 10): no intervention	10 weeks/20 sessions; 2 sets of 10–20 reps or 10–60 s; 30 min; N/A	Quadruped, dying bugs, bridging, static plank	Static back endurance test	STU-pp: 30.3 % (SMD_w_ 0.63) CON-pp: −29.1 % (SMD_w_ −0.55) STU-CON: SMD_b_ 2.09 (95 % CI 0.95, 3.22)
					Side bridge test	STU-pp: 57.0 % (SMD_w_ 0.66) CON-pp: 23.4 % (SMD_w_ 0.37) STU-CON: SMD_b_ 0.38 (95 % CI −0.50, 1.27)
Cowley et al. [43]	14 F; 20–23 years; untrained	STU (*n* = 7): upper-body strength exercises on unstable surface (i.e. Swiss ball) STS (*n* = 7): upper-body strength exercises on stable surface	3 weeks/7 sessions; 3 sets of 3–5 reps; N/A; 85–90 % 1RM	Chest press	1RM bench press test	STU-pp: 15.7 % (SMD_w_ 2.74) STS-pp: 18.8 (SMD_w_ 2.65) STU-STS: SMD_b_ −0.12 (95 % CI −1.17, 0.93)
					YMCA bench press test	STU-pp: 13.0 % (SMD_w_ 1.43) STS-pp: 25.2 % (SMD_w_ 1.93) STU-STS: SMD_b_ −0.45 (95 % CI −1.52, 0.61)
					Abdominal power test (front, side)	Front: STU-pp: 4.6 % (SMD_w_ 0.29) STS-pp: 22.5 % (SMD_w_ 1.73) STU-STS: SMD_b_ 0.39 (95 % CI −0.67, 1.45) Side: STU-pp: −5.6 % (SMD_w_ −0.42) STS-pp: −2.9 % (SMD_w_ −0.23) STU-STS: SMD_b_ 0.69 (95 % CI −0.40, 1.78)
Cressey et al. [39]	19 M; 18–23 years; trained; soccer	STU (*n* = 10): soccer training + lower-body strength exercises on unstable surface (i.e. Swiss ball) STS (*n* = 9): soccer training + lower-body strength exercises on stable surface	10 weeks/27 sessions; 2–5 sets of 5–15 reps; N/A; 55 % 1RM	Deadlift, dumbbell lunge, barbell press, dumbbell row, side bridge	CMJ	STU-pp: 0 % (SMD_w_ 0) STS-pp: 2.5 % (SMD_w_ 0.22) STU-STS: SMD_b_ −0.43 (95 % CI −1.34, 0.48)
					Bounce DJ	STU-pp: 0.8 % (SMD_w_ 0.11) STS-pp: 3.3 % (SMD_w_ 0.26) STU-STS: SMD_b_ −0.41 (95 % CI −1.32, 0.50)
Kibele and Behm [13]	40 (28 M, 12 F); 23 ± 4 years; untrained	STU (*n* = 20): lower-/upper-body strength exercises on unstable surface (i.e. Swiss ball) STS (*n* = 20): lower-/upper-body strength exercises on stable surface	7 weeks/14 sessions; 3–5 sets of 6–15 reps; N/A; 50–75 % 1RM	Both groups: squat, vertical jump, pulldown, butterfly, bench press; STS only: trunk stabilization exercises	MIMS leg extension	STU-pp: 8.2 % (SMD_w_ 0.26) STS-pp: 10.9 % (SMD_w_ 0.44) STU-STS: SMD_b_ −0.03 (95 % CI −0.65, 0.59)
					SLJ	STU-pp: 4.5 % (SMD_w_ 0.33) STS-pp: 4.5 % (SMD_w_ 0.33) STU-STS: SMD_b_ 0 (95 % CI −0.62, 0.62)
					Left and right leg hop	Left leg: STU-pp: 6.7 % (SMD_w_ 0.43) STS-pp: 4.3 % (SMD_w_ 0.20) STU-STS: SMD_b_ 0.27 (95 % CI −0.35, 0.89) Right leg: STU-pp: 6.7 % (SMD_w_ 0.43) STS-pp: 0 % (SMD_w_ 0) STU-STS: SMD_b_ 0.28 (95 % CI −0.35, 0.90)
					Sit-ups	STU-pp: 8.9 % (SMD_w_ 0.57) STS-pp: 2.6 % (SMD_w_ 0.23) STU-STS: SMD_b_ −0.17 (95 % CI −0.80, 0.45)
					2-legged stance with eyes opened and on foam ground	STU-pp: 45.2 % (SMD_w_ 2.07) STS-pp: 44.5 % (SMD_w_ 2.03) STU-STS: SMD_b_ 0.29 (95 % CI −0.34, 0.91)
					6-m backward walking	STU-pp: 15.2 % (SMD_w_ 0.75) STS-pp: 9.1 % (SMD_w_ 0.54) STU-STS: SMD_b_ 0.18 (95 % CI −0.44, 0.80)
Sato and Mokha [41]	20 (10 M, 10 F); 37 ± 9 years; trained; running	STU (*n* = 12): running training + core strength exercises on unstable surface (i.e. Swiss ball) CON (*n* = 8): running training only	6 weeks/24 sessions; 2–3 sets of 10–15 reps; N/A; N/A	Abdominal crunch, back extension, supine opposite arm/leg raise, hip raise, Russian twist	SEBT (composite score for both legs)	STU-pp: 11.0 % (SMD_w_ 0.82) CON-pp: 5.1 % (SMD_w_ 0.39) STU-CON: SMD_b_ 0.40 (95 % CI −0.50, 1.31)
Sparkes and Behm [22]	18 (10 M, 8 F); 18–30 years; untrained	STU (*n* = 9): lower-/upper-body strength exercises on unstable surface (i.e. DynaDisc^®^, Swiss ball) STS (*n* = 9): lower-/upper-body strength exercises on stable surface	8 weeks/24 sessions; 2 sets of 10 reps; N/A; 10RM	Bench press, dumbbell press, dumbbell row, lat cable pulldown, leg press, leg curl, calf raise	MIMS bench press	STU-pp: 8.4 % (SMD_w_ 0.19) STS-pp: 7.4 % (SMD_w_ 0.22) STU-STS: SMD_b_ −0.30 (95 % CI −1.23, 0.63)
					3RM squat	STU-pp: 11.8 % (SMD_w_ 0.52) STS-pp: 18.4 % (SMD_w_ 0.43) STU-STS: SMD_b_ 0.47 (95 % CI −0.47, 1.41)
					Medicine ball throw	STU-pp: 5.1 % (SMD_w_ 0.26) STS-pp: 6.3 % (SMD_w_ 0.75) STU-STS: SMD_b_ −2.34 (95 % CI −3.61, −1.08)
					2-legged stance with eyes opened and on foam ground	STU-pp: 40.0 % (SMD_w_ 0.82) STS-pp: 76.2 % (SMD_w_ 1.54) STU-STS: SMD_b_ −0.24 (95 % CI −1.17, 0.68)
Chulvi-Medrano et al. [45]	30 M; 25 ± 3 years; trained; N/A	STU I (*n* = 10): upper-body strength exercises on unstable surface (i.e. T-Bow^®^) STU II (*n* = 10): upper-body strength exercises on unstable surface (i.e. BOSU^®^ ball) STS (*n* = 10): upper-body strength exercises on stable surface	8 weeks/16 sessions; 3 sets of 10 reps; N/A; ≥7 on the OMNI-R (0–10) Scale	Push-up	1RM bench press	STU I-pp: 4.3 % (SMD_w_ 0.38) STU II-pp: 5.0 % (SMD_w_ 0.39) STS-pp: 0.5 % (SMD_w_ 0.04) STU I-STS: SMD_b_ 0.31 (95 % CI −0.58, 1.19) STU II-STS: SMD_b_ −0.17 (95 % CI −1.05, 0.71)
					Push-ups	STU I-pp: 13.5 % (SMD_w_ 0.48) STU II-pp: 14.4 % (SMD_w_ 0.43) STS-pp: 4.8 % (SMD_w_ 0.16) STU I-STS: SMD_b_ 0.75 (95 % CI −0.16, 1.66) STU II-STS: SMD_b_ 0.03 (95 % CI −0.84, 0.91)
Cug et al. [46]	60 (36 M, 24 F); 23 ± 2 years; untrained	STU (*n* = 43): lower-/upper-body strength exercises on unstable surface (i.e. Swiss ball) CON (*n* = 17): no intervention	10 weeks/30 sessions; 2–3 sets of 6–14 reps (30–60 s each); 30 min; N/A	Abdominal crunch, back extension, supine hamstring curl, squat, standing and kneeling on the Swiss ball	Isokinetic trunk flexor/extensor strength	Trunk flexion: STU-pp: 18.1 % (SMD_w_ 0.80) CON-pp: −0.4 % (SMD_w_ −0.02) STU-CON: SMD_b_ 0.68 (95 % CI 0.11, 1.26) Trunk extension: STU-pp: 23.6 % (SMD_w_ 0.95) CON-pp: −6.8 % (SMD_w_ −0.19) STU-CON: SMD_b_ 0.96 (95 % CI 0.37, 1.54)
Marinkovic et al. [32]	50 (M N/A, F N/A); 21 ± 1 years; untrained	STU (*n* = 25): lower-/upper-body strength exercises on unstable surface (i.e. BOSU^®^ ball, Swiss ball) STS (*n* = 25): lower-/upper-body strength exercises on stable surface	8 weeks/16 sessions; 2 sets of 10 reps; N/A; 50 % 1RM	Bench press, barbell squat	1RM bench press	STU-pp: 3.2 % (SMD_w_ 0.15) STS-pp: 2.7 % (SMD_w_ 0.17) STU-STS: SMD_b_ −0.03 (95 % CI −0.58, 0.53)
					1RM barbell squat	STU-pp: 5.4 % (SMD_w_ 0.35) STS-pp: 2.1 % (SMD_w_ 0.12) STU-STS: SMD_b_ 0.21 (95 % CI −0.35, 0.77)
Oberacker et al. [40]	19 F; 19 ± 1 years; trained; soccer	STU (*n* = 9): soccer training + lower-/upper-body strength exercises on unstable surface (i.e. BOSU^®^ ball) STS (*n* = 10): soccer training + lower-/upper-body strength exercises on stable surface	5 weeks/15 sessions; 4 reps; N/A; 4–6RM	Push press, back squat, hang clean, lunge, bent over row, bench press, deadlift	CMJ	STU-pp: −4.3 % (SMD_w_ −0.50) STS-pp: 17.4 % (SMD_w_ 1.33) STU-STS: SMD_b_ −1.34 (95 % CI −2.36, −0.32)
Premkumar et al. [33]	30 F; 19–25 years; untrained	STU (*n* = 15): upper-body strength exercises on unstable surface (i.e. Swiss ball) STS (*n* = 15): upper-body strength exercises on unstable surface	2 weeks/14 sessions; 5 reps; N/A; 100 % 1RM	Barbell chest press	1RM bench press	STU-pp: 38.3 % (SMD_w_ 5.54) STS-pp: 21.3 % (SMD_w_ 1.46) STU-STS: SMD_b_ 2.35 (95 % CI 1.39, 3.31)
					Sit-ups	STU-pp: 22.5 % (SMD_w_ 4.01) STS-pp: 25.5 % (SMD_w_ 4.59) STU-STS: SMD_b_ 1.32 (95 % CI 0.52, 2.12)
Sukalinggam et al. [23]	42 (sex N/A); 24 ± 3 years; untrained	STU (*n* = 14): core strength exercises on unstable surface (i.e. Swiss ball) STS (*n* = 14): core strength exercises on stable surface CON (*n* = 14): no intervention	6 weeks/12 sessions; 1–3 sets of 20–60 reps or 60–120 s; 30–40 min; N/A	Crunch, supine leg lift, back extension, reverse back extension, supine rotation, side bend, seated balance, core endurance	1RM abdominal curl	STU-pp: 27.4 % (SMD_w_ 0.43) STS-pp: 8.5 % (SMD_w_ 0.16) CON-pp: −4.7 % (SMD_w_ −0.13) STU-STS: SMD_b_ 0.50 (95 % CI −0.26, 1.25) STU-CON: SMD_b_ 0.88 (95 % CI 0.10, 1.66)
Mate-Munoz et al. [47]	30 M; 22 ± 2 years; untrained	STU (*n* = 12): lower-/upper-body strength exercises on unstable surface (i.e. BOSU^®^ ball) STS (*n* = 12): lower-/upper-body strength exercises on stable surface CON (*n* = 12): no intervention	7 weeks/21 sessions; 3 sets of 15 reps; 45–65 min; ≥5 on the Borg (CR-10) Scale	Back cable pulldown, lunge dumbbell, bench press, shoulder press, power snatch, biceps curl, triceps extension, back squat, seated row	1RM bench press	STU-pp: 4.7 % (SMD_w_ 0.45) STS-pp: 4.4 % (SMD_w_ 0.22) CON-pp: −0.9 % (SMD_w_ −0.04) STU-STS: SMD_b_ −0.08 (95 % CI −0.88, 0.72) STU-CON: SMD_b_ 0.01 (95 % CI −0.79, 0.81)
					1RM back squat	STU-pp: 13.0 % (SMD_w_ 0.78) STS-pp: 12.6 % (SMD_w_ 0.41) CON-pp: −0.6 % (SMD_w_ −0.04) STU-STS: SMD_b_ −0.13 (95 % CI −0.93, 0.67) STU-CON: SMD_b_ 1.00 (95 % CI 0.14, 1.86)
					CMJ	STU-pp: 17.7 % (SMD_w_ 1.00) STS-pp: 15.2 % (SMD_w_ 0.74) CON-pp: 0.3 % (SMD_w_ 0.02) STU-STS: SMD_b_ −0.32 (95 % CI −1.13, 0.48) STU-CON: SMD_b_ 0.61 (95 % CI −0.21, 1.43)
					SJ	STU-pp: 22.1 % (SMD_w_ 1.23) STS-pp: 20.1 % (SMD_w_ 0.92) CON-pp: 0.7 % (SMD_w_ 0.04) STU-STS: SMD_b_ −0.35 (95 % CI −1.15, 0.46) STU-CON: SMD_b_ 0.64 (95 % CI −0.19, 1.46)
Kibele et al. [44]	33 M; 24 ± 4 years; untrained	STU (*n* = 20): plyometric exercises on unstable surface (e.g. balance pad) STS (*n* = 13): plyometric exercises on stable surface	7 weeks/14 sessions; 3 sets of 5–10 reps; 40 min; 50 % 1RM	Bilateral CMJ, DJ; bilateral hurdle jumps, high-bar squats	MIMS leg extension	STU-pp: 11.7 % (SMD_w_ 0.57) STS-pp: 14.3 % (SMD_w_ 0.78) STU-STS: SMD_b_ 0.20 (95 % CI −0.50, 0.90)
					CMJ	STU-pp: 13.6 % (SMD_w_ 1.00) STS-pp: 5.3 % (SMD_w_ 0.49) STU-STS: SMD_b_ −0.35 (95 % CI −1.05, 0.35)
					Hurdle DJ	STU-pp: 9.5 % (SMD_w_ 0.74) STS-pp: 4.3 % (SMD_w_ 0.31) STU-STS: SMD_b_ −0.44 (95 % CI −1.14, 0.27)
					20-m left–right hop	STU-pp: 0 % (SMD_w_ 0) STS-pp: 0 % (SMD_w_ 0) STU-STS: SMD_b_ 0 (95 % CI −0.70, 0.70)
					Standing stork test	STU-pp: 12.4 % (SMD_w_ 0.16) STS-pp: −12.1 % (SMD_w_ −0.16) STU-STS: SMD_b_ −0.40 (95 % CI −1.10, 0.31)
					Forward/backward walking	STU-pp: 18.4 % (SMD_w_ 1.00) STS-pp: 17.9 % (SMD_w_ 0.88) STU-STS: SMD_b_ 0.20 (95 % CI −0.50, 0.90)
Old adults						
Chulvi-Medrano et al. [48]	28 F; ≥65 years; untrained	STU (*n* = 18): lower-body strength exercises on unstable surface (i.e. T-Bow^®^) CON (*n* = 10): no intervention	8 weeks/16 sessions; 1–3 sets of 12 reps (30 s each); 30 min; N/A	Squat, lateral and frontal swing, lunge, plantarflexion	1-legged stance with eyes opened and on firm ground (dominant leg)	STU-pp: 35.2 % (SMD_w_ 3.47) CON-pp: −5.8 % (SMD_w_ −0.62) STU-CON: SMD_b_ 2.86 (95 % CI 1.74, 3.97)
					8-foot Up and Go test	STU-pp: 12.7 % (SMD_w_ 2.64) CON-pp: 6.5 % (SMD_w_ 0.74) STU-CON: SMD_b_ 2.56 (95 % CI 1.50, 3.62)
Seo et al. [34]	78 F; 72 ± 8 years; untrained	STU (*n* = 38): lower-/upper-body strength exercises on unstable surface (i.e. Swiss ball) CON (*n* = 40): no intervention	12 weeks/24 sessions; 3–5 sets of 2–10 reps; 30 min; Borg Scale (no report of scaling type)	Bridging, sit-up, back extension, pelvic rotation, hip adduction, leg raise, knee flexion/extension, bounce, ball push	Sit-to-stand test	STU-pp: 8.6 % (SMD_w_ 0.36) CON-pp: −1.4 % (SMD_w_ −0.06) STU-CON: SMD_b_ 0.20 (95 % CI −0.25, 0.64)
					Arm curls	STU-pp: 2.5 % (SMD_w_ 0.16) CON-pp: −2.2 % (SMD_w_ −0.11) STU-CON: SMD_b_ 0.18 (95 % CI −0.27, 0.62)
					1-legged stance with eyes closed and on firm ground	STU-pp: 10.3 % (SMD_w_ 0.33) CON-pp: −0.7 % (SMD_w_ −0.03) STU-CON: SMD_b_ −0.09 (95 % CI −0.53, 0.36)
					TUG	STU-pp: 8.9 % (SMD_w_ 0.41) CON-pp: 0.8 % (SMD_w_ 0.04) STU-CON: SMD_b_ 0.66 (95 % CI 0.20, 1.11)
Granacher et al. [18]	32 (15 M, 17 F); 63–80 years; untrained	STU (*n* = 16): core strength exercises on unstable surface (i.e. Swiss ball, balance pad) CON (*n* = 16): no intervention	9 weeks/18 sessions; 3–4 sets of 15–20 reps or 15–20 s; 45 min; N/A	Curl-up, side bridge, quadruped	MIMS trunk flexors/extensors/lateral flexors (left, right)/rotators (left, right)	Flexion: STU-pp: 33.6 % (SMD_w_ 0.78) CON-pp: 1.0 % (SMD_w_ 0.02) STU-CON: SMD_b_ 0.44 (95 % CI −0.26, 1.14) Extension: STU-pp: 21.5 % (SMD_w_ 0.49) CON-pp: −0.3 % (SMD_w_ −0.01) STU-CON: SMD_b_ 0.60 (95 % CI −0.11, 1.31) Lateral flexion left: STU-pp: 53.1 % (SMD_w_ 1.12) CON-pp: 7.2 % (SMD_w_ 0.17) STU-CON: SMD_b_ 0.40 (95 % CI −0.30, 1.10) Lateral flexion right: STU-pp: 48.1 % (SMD_w_ 1.08) CON-pp: −1.6 % (SMD_w_ −0.04) STU-CON: SMD_b_ 0.64 (95 % CI −0.08, 1.35) Rotation left: STU-pp: 41.8 % (SMD_w_ 0.79) CON-pp: −1.5 % (SMD_w_ −0.03) STU-CON: SMD_b_ 0.33 (95 % CI −0.37, 1.03) Rotation right: STU-pp: 38.4 % (SMD_w_ 0.66) CON-pp: 18.0 % (SMD_w_ 0.32) STU-CON: SMD_b_ −0.12 (95 % CI −0.81, 0.58)
					10-m walk test	STU-pp: 8.6 % (SMD_w_ 0.85) CON-pp: 0.4 % (SMD_w_ 0.03) STU-CON: SMD_b_ 0.63 (95 % CI −0.08, 1.34)
					TUG	STU-pp: 4.2 % (SMD_w_ 0.40) CON-pp: −4.3 % (SMD_w_ −0.50) STU-CON: SMD_b_ 0.96 (95 % CI 0.23, 1.70)
					FRT	STU-pp: 20.4 % (SMD_w_ 1.27) CON-pp: 4.6 % (SMD_w_ 0.26) STU-CON: SMD_b_ 1.04 (95 % CI 0.30, 1.79)

*CI* confidence interval*, CMJ* countermovement jump, *CON* control group, *DJ* drop jump, *F* female, *FRT* functional reach test, *lat* lateral, *M* male, *MIMS* maximal isometric muscle strength, *N/A* not available, *pp* pre- to post-test change, *reps* repetitions, *RM* repetition maximum, *SEBT* Star Excursion Balance test, *SJ* squat jump, *SLJ* standing long jump, SMD_*b*_ between-subject standardized mean difference (i.e. unstable versus stable/control comparison), SMD_*w*_ within-subject standardized mean difference (i.e. pre- versus post-test comparison), *STS* strength training on stable surfaces, *STU* strength training on unstable surfaces, *TUG* Timed Up and Go test, *YBT* Y Balance test

Four studies (1 × STU versus CON, 3 × STU versus STS) examined the effects of STU in adolescents [35–38]. Three of them were conducted with trained subjects [35, 36, 38] and one with untrained subjects [37]. A total of 106 adolescents participated in the four studies, and 52 of those received STU. The sample size of the STU groups ranged from eight to 18 subjects. STU protocols that were conducted in adolescents involved core strength training (e.g. supine leg bridge, side bridge) and plyometric training on stable surfaces (e.g. CMJ, drop jump [DJ]) or unstable surfaces (e.g. balance pad, Dynair^®^ Cushion, Swiss ball). To evaluate the effects of STU in adolescents, one study [36] used a test for assessment of maximal isometric muscle strength (MIMS) (i.e. MIMS trunk flexors/extensors), two studies [35, 37] used tests for assessment of strength endurance (i.e. Bourban trunk muscle strength test, prone stabilization core stability test) and three studies [36–38] used tests for assessment of muscle power [i.e. CMJ, DJ, standing long jump (SLJ), Multiple 5 Bounds test]. Furthermore, two studies [37, 38] tested for static balance (e.g. one-legged stance time), as well as dynamic balance [i.e. reach distance in the Star Excursion Balance test (SEBT), *Y* balance test]. The training period for STU ranged from 6 to 9 weeks, with a total of 12–27 training sessions. The numbers of sets and repetitions per exercise ranged from 2 to 5 and from 5 to 25, respectively. The duration per exercise lasted between 15 and 50 s. Lastly, the duration of a single training session ranged from 25 to 35 minutes. None of the studies provided specific information on (perceived) training intensity (e.g. the Borg Scale). General information on progression during STU was given in terms of an increase in the number of sets, repetitions or duration per exercise. Additionally, training progression was achieved by increasing the difficulty level of the respective exercises (i.e. modulation of the lever length or type of muscle action; increase of drop, jump or hurdle height; reduction of base of support; addition of opposite limb movements).

Further, our systematic search revealed 15 studies (6 × STU versus CON, 12 × STU versus STS) that investigated the effects of STU on measures of muscle strength, power and balance in young adults. Three of them included trained participants [39–41] and 12 included untrained participants [13, 22, 23, 31–33, 42–47]. A total of 480 young adults participated in the 15 studies, and 246 of those received STU. The sample size of the STU groups ranged from 7 to 43 subjects. STU protocols that were conducted in young adults involved core strength training (e.g. bridging, static plank) and lower-body strength exercises (e.g. dead lift, leg press), as well as upper-body strength exercises (e.g. chest press, dumbbell row), on unstable surfaces (e.g. a balance pad, BOSU^®^ ball, DynaDisc^®^, Resist-A-Ball^®^, Swiss ball, T-Bow^®^). To evaluate STU effects in young adults, 11 studies [13, 22, 23, 31–33, 43–47] used a test for assessment of maximal muscle strength (i.e. 1RM abdominal curl, 1RM barbell/back squat, 1RM bench press, double leg-lowering test, leg extension, YMCA bench press test), five studies [13, 31, 33, 42, 45] applied tests for assessment of muscular endurance (i.e. back extension, push-ups, side bridge test, sit-ups, static back endurance test, trunk flexion) and seven studies [13, 22, 39, 40, 43, 44, 47] used tests to determine muscle power (i.e. abdominal power test, CMJ, DJ, leg hop, medicine ball throw, SLJ). Furthermore, three studies [13, 22, 44] tested for static balance (i.e. two-legged stance, standing stork test) and three studies [13, 41, 44] tested for dynamic balance (i.e. 6-m backward walking, forward/backward walking, SEBT). The training periods for STU in young adults ranged from 2 to 10 weeks, with a total of 7–30 training sessions. The numbers of sets and repetitions per exercise ranged from 1 to 5 and from 3 to 60, respectively. The duration of single exercises lasted between 10 and 120 s, and the duration of a single training session lasted between 30 and 65 min. Information on (perceived) training intensity during STU in young adults was provided in nine studies and ranged from 50 to 100 % 1RM, ≥5 on the Borg Scale (CR-10) and ≥7 on the OMNI-R Scale (0–10). General information on progression during STU was given in terms of an increase in the number of sets, the number of repetitions or the duration per exercise. Additionally, the difficulty level of STU exercises progressed over the course of the study by applying additional loads and by increasing drop, jump and hurdle height [40, 44].

Three studies (3 × STU versus CON) examined the effects of STU in old adults [18, 34, 48]. All of them included untrained subjects. A total of 128 seniors participated in the three studies, and 72 of those received STU. The sample sizes of the STU groups ranged from 16 to 38 subjects. STU protocols in old adults involved core strength training (e.g. quadruped, side bridge) and lower-body strength exercises (e.g. squat, lunge), as well as upper-body strength exercises (e.g. back extension, sit-up), on unstable surfaces (e.g. a balance pad, Swiss ball, T-Bow^®^). To evaluate the effects of STU in old adults, one study [18] used a test to determine MIMS (i.e. MIMS trunk flexors/extensors/lateral flexors/rotators) and another study [34] applied tests for assessment of muscular power (i.e. sit-to-stand test, arm curls). Furthermore, two studies [34, 48] tested for static balance (i.e. one-legged stance) and three studies [18, 34, 48] tested for dynamic balance (i.e. 10-m walk test, functional reach test, Timed Up and Go test, 8-Foot Up and Go test). STU training periods in old adults lasted between 8 and 12 weeks, with a total of 16–24 training sessions. The applied numbers of sets and repetitions per exercise ranged between one and five and between 2 and 20, respectively. A single exercise lasted between 15 and 20 s, and the duration of a single training session ranged from 30 to 45 min. Information on perceived training intensity during STU in old adults was provided in one study [34] using the Borg Scale (≥7) (no report of scaling type). General information on progression during STU was given in terms of an increase in the numbers of sets, repetitions or duration per exercise. Additionally, training progression was achieved by increasing the difficulty level of the respective STU exercise (i.e. modulation of lever length, range of motion or movement velocity, reduction of base of support).

### Methodological Quality of the Included Trials

In general, the quality of the included studies was rather low, with mean PEDro scores of 5.8, 4.0 and 5.0 for studies examining adolescents, young adults and old adults, respectively. The predetermined cut-off score of ≥6 on the PEDro Scale was achieved by three out of four studies in adolescents [36–38], none out of 15 studies in young adults and two out of three studies in old adults [18, 48] (Table 2).

**Table 2:** Physiotherapy Evidence Database (PEDro) scores of the reviewed studies

Authors	Eligibility criteria	Randomized assignation	Blinded assignation	Group homogeneity	Blinded subjects	Blinded coaches	Blinded investigators	Dropout <15 %	Intention-to-treat	Group comparisons	Point and variability measures	Total PEDro score
Adolescents												
Stanton et al. [35]	−	−	−	+	−	−	−	+	+	+	+	5
Granacher et al. [37]	+	+	−	+	−	−	−	+	+	+	+	6
Prieske et al. [36]	+	+	−	+	−	−	−	+	+	+	+	6
Granacher et al. [38]	+	+	−	+	−	−	−	+	+	+	+	6
Young adults												
Stanforth et al. [31]	−	−	−	+	−	−	−	−	+	+	+	4
Carter et al. [42]	+	+	−	−	−	−	−	+	+	+	+	5
Cowley et al. [43]	−	−	−	−	−	−	−	−	+	+	+	3
Cressey et al. [39]	+	+	−	+	−	−	−	−	+	+	+	5
Kibele and Behm [13]	−	+	−	−	−	−	−	−	+	+	+	4
Sato and Mokha [41]	−	+	−	−	−	−	−	−	−	+	+	3
Sparkes and Behm [22]	+	+	−	+	−	−	−	−	+	+	+	5
Chulvi-Medrano et al. [45]	+	+	−	−	−	−	−	−	+	+	+	4
Cug et al. [46]	+	−	−	+	−	−	−	−	+	+	+	4
Marinkovic et al. [32]	−	−	−	−	−	−	−	−	+	+	+	3
Oberacker et al. [40]	+	−	−	−	−	−	−	−	+	+	+	3
Premkumar et al. [33]	−	+	−	+	−	−	−	−	+	+	+	5
Sukalinggam et al. [23]	−	+	−	−	−	−	−	−	+	+	+	4
Mate-Munoz et al. [47]	−	+	−	−	−	−	−	−	+	+	+	4
Kibele et al. [44]	−	+	−	−	−	−	−	−	+	+	+	4
Old adults												
Chulvi-Medrano et al. [48]	+	+	−	+	−	−	−	+	+	+	+	6
Seo et al. [34]	−	−	−	−	−	−	−	−	+	+	+	3
Granacher et al. [18]	+	+	−	+	−	−	−	+	+	+	+	6

The eligibility criteria have to be excluded for calculation of the total PEDro score; + indicates a ‘yes’ score; − indicates a ‘no’ score

### Effectiveness of Strength Training on Unstable Surfaces Versus Control Condition

**Fig. 2:**
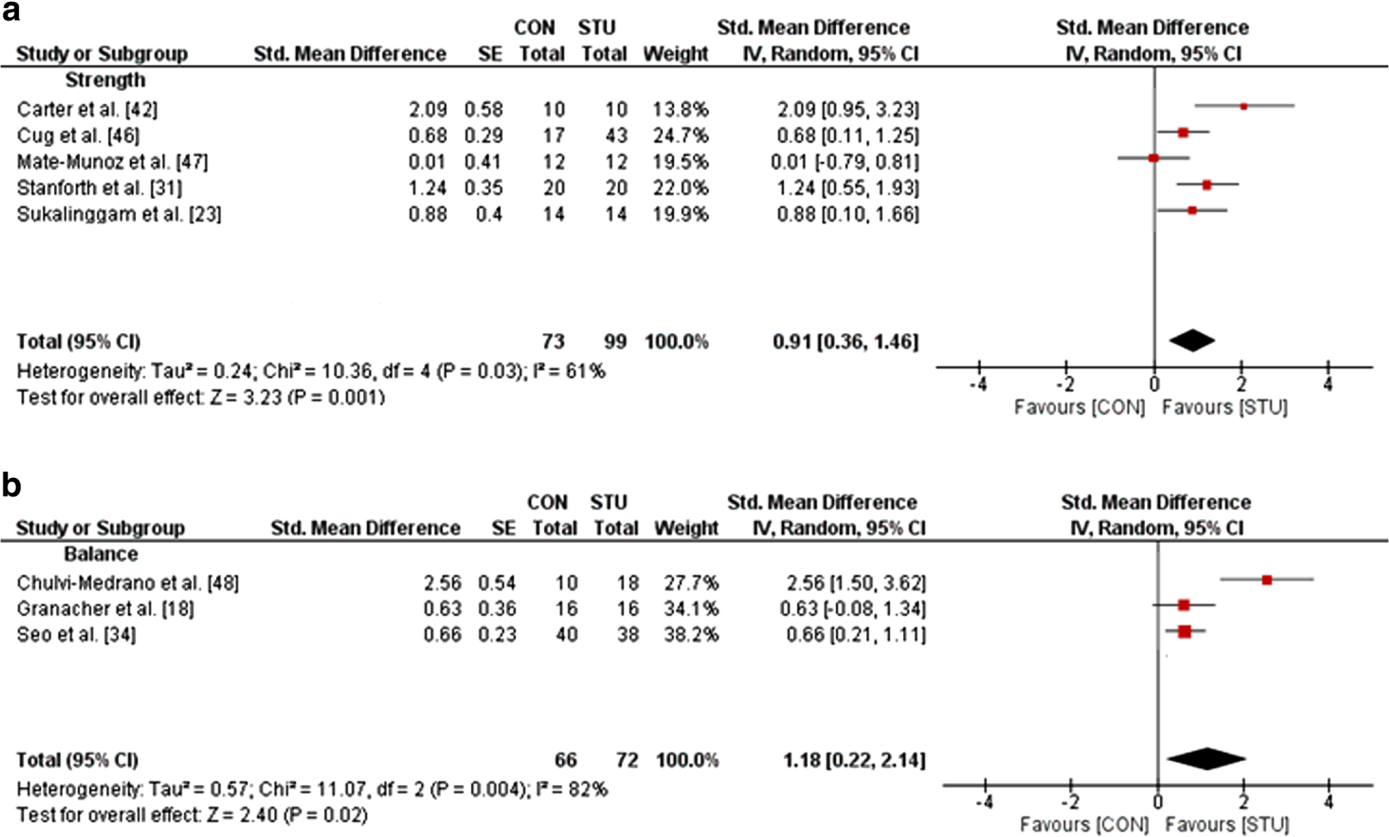
Effects of strength training on unstable surfaces (STU) versus control condition (CON; i.e. no training or regular training only) on measures of strength (**a**) and balance (**b**). *CI* confidence interval, *df* degrees of freedom, *IV* inverse variance, *SE* standard error, *Std.* standard

**Fig. 3:**
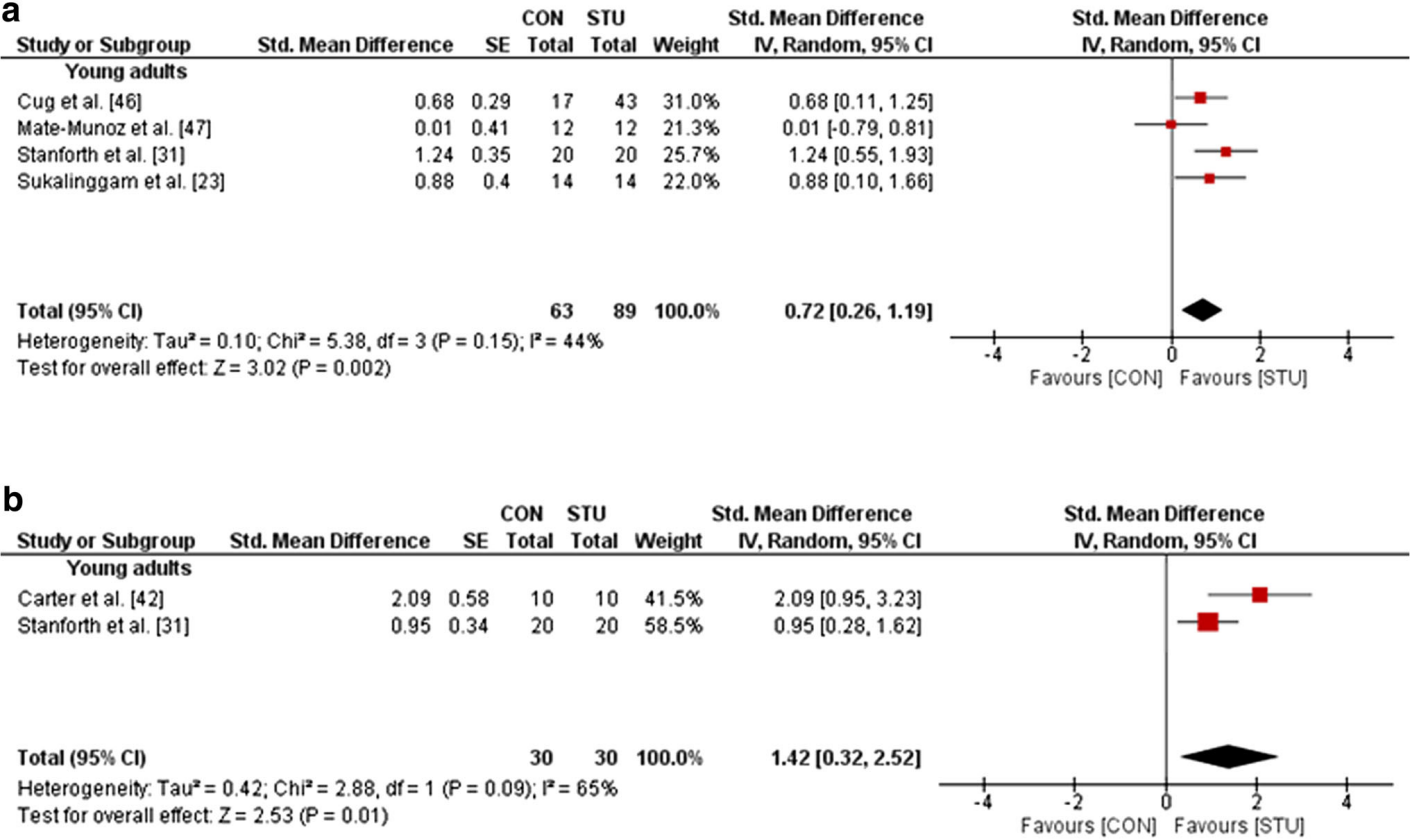
Effects of strength training on unstable surfaces (STU) versus control condition (CON; i.e. no training or regular training only) on measures of maximal strength (**a**) and strength endurance (**b**) in healthy young adults. *CI* confidence interval, *df* degrees of freedom, *IV* inverse variance, *SE* standard error, *Std.* standard

One study in adolescents [35], six studies in young adults [23, 31, 41, 42, 46, 47], and three studies in old adults [18, 34, 48], but no studies in children and middle-aged adults, examined the effects of STU compared with CON (i.e. no training or regular training only) on measures of strength, power and balance (Table 1). A general forest plot for measures of muscle strength and balance is presented in Fig. 2a, b. Our analyses revealed large effects of STU on muscle strength (mean SMD_b_ = 0.91, *I*^2^ = 61 %, *χ*^2^ = 10.36, degrees of freedom (*df*) = 4, *p* = 0.03; five studies [23, 31, 42, 46, 47]; Fig. 2a) and balance outcomes (mean SMD_b_ = 1.18, *I*^2^ = 82 %, *χ*^2^ = 11.07, *df* = 2, *p* = 0.004; three studies [18, 34, 48]; Fig. 2b). Additionally, our item-specific analyses revealed that STU produced medium effects on variables of maximal strength in young adults (mean SMD_b_ = 0.72, *I*^2^ = 44 %, *χ*^2^ = 5.38, *df* = 3, *p* = 0.15; four studies [23, 31, 46, 47]; Fig. 3a) and, depending on the analysed variable, no effects (i.e. maximal strength of the trunk rotators right) to medium effects [i.e. maximal strength of the trunk flexors, rotators, lateral flexors (left/right), lateral rotators (left)] in old adults (SMD_b_ = −0.12 to 0.64; one study [18]). Additionally, our analysis revealed large effects of STU on measures of strength endurance in adolescents (SMD_b_ = 3.86; one study [35]) and in young adults (mean SMD_b_ = 1.42, *I*^2^ = 65 %, *χ*^2^ = 2.88, *df* = 1, *p* = 0.09; two studies [31, 42]; Fig. 3b). However, only a small effect was found in old adults (SMD_b_ = 0.18; one study [34]). Furthermore, medium effects on muscle power were detected for STU as compared with CON in young adults (SMD_b_ = 0.61–0.64; one study [47]) and a small effect in old adults (SMD_b_ = 0.20; one study [34]).

**Fig. 4:**
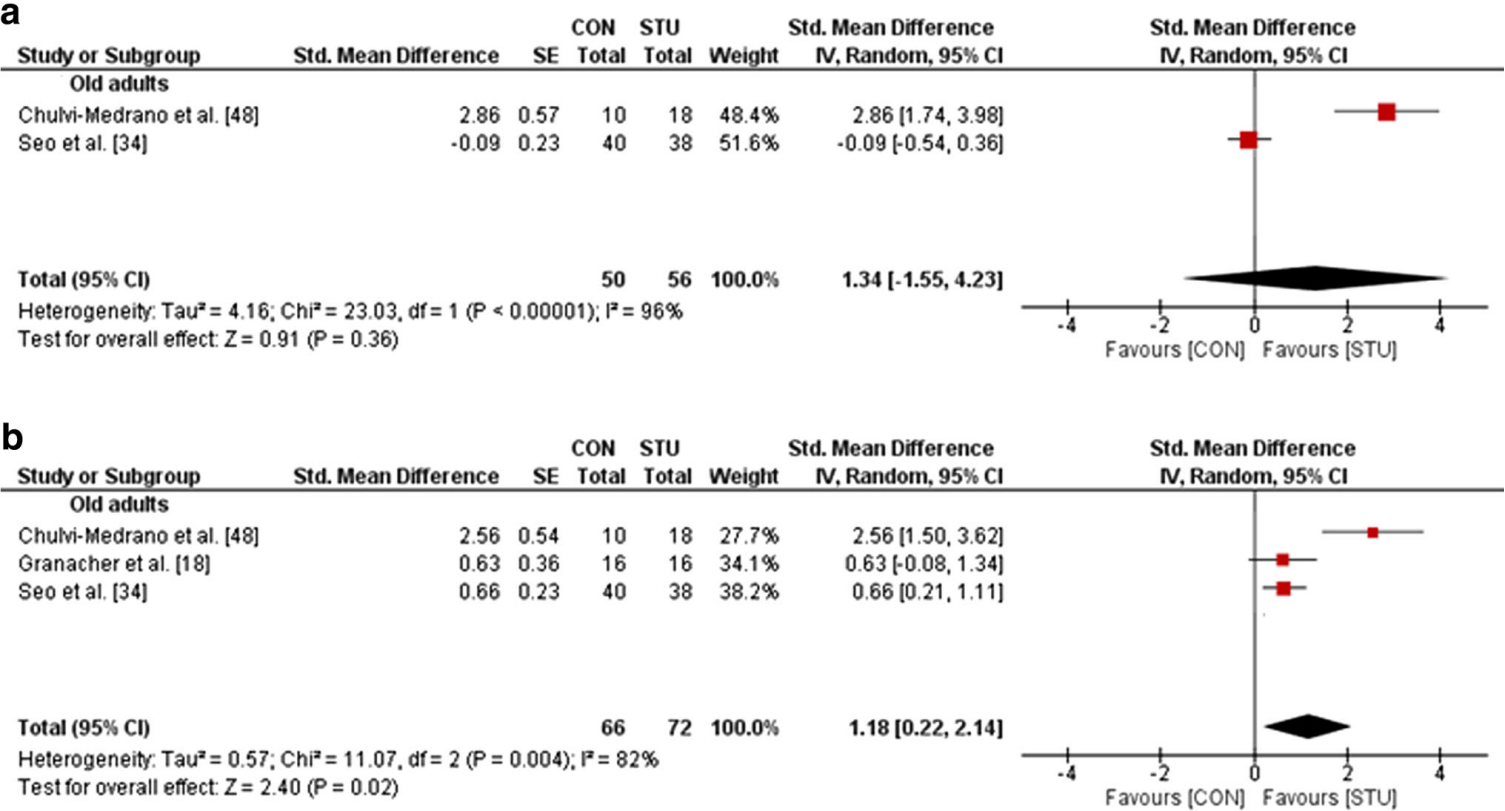
Effects of strength training on unstable surfaces (STU) versus control condition (CON; i.e. no training or regular training only) on measures of static balance (**a**) and dynamic balance (**b**) in healthy old adults. *CI* confidence interval, *df* degrees of freedom, *IV* inverse variance, *SE* standard error, *Std.* standard

In terms of static balance, STU yielded large effects in old adults (mean SMD_b_ = 1.34, *I*^2^ = 96 %, *χ*^2^ = 23.03, *df* = 1, *p* < 0.001; two studies [34, 48]; Fig. 4a) in comparison with CON. Lastly, a small effect was detected for measures of dynamic balance in young adults (SMD_b_ = 0.40; one study [41]) and large effects in old adults (mean SMD_b_ = 1.18, *I*^2^ = 82 %, *χ*^2^ = 11.07, *df* = 2, *p* = 0.004; three studies [18, 34, 48]; Fig. 4b).

### Effectiveness of Strength Training on Unstable Versus Stable Surfaces

**Fig. 5:**
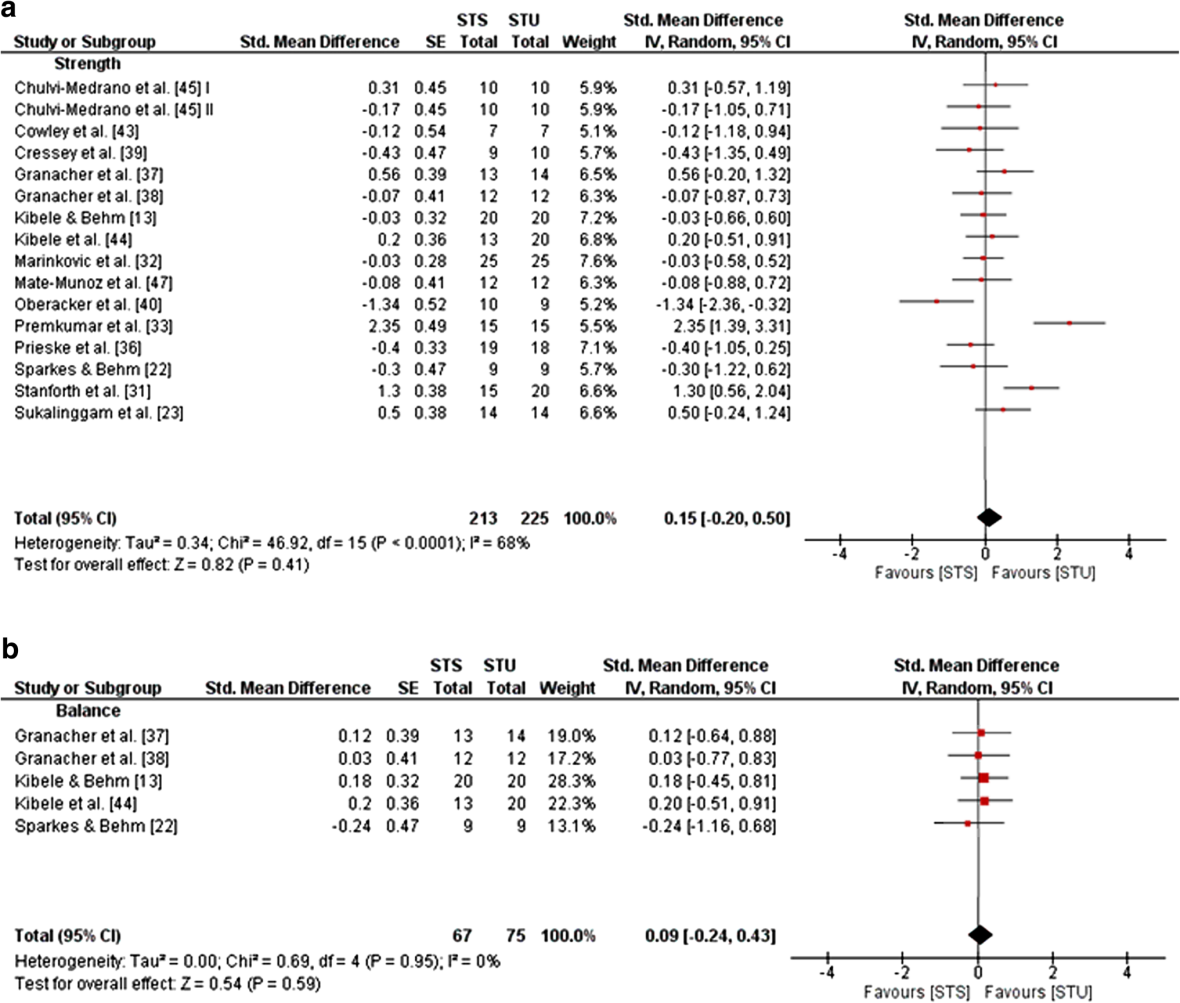
Effects of strength training on unstable surfaces (STU) versus stable surfaces (STS) on measures of strength (**a**) and balance (**b**). *CI* confidence interval, *df* degrees of freedom, *IV* inverse variance, *SE* standard error, *Std.* standard

**Fig. 6:**
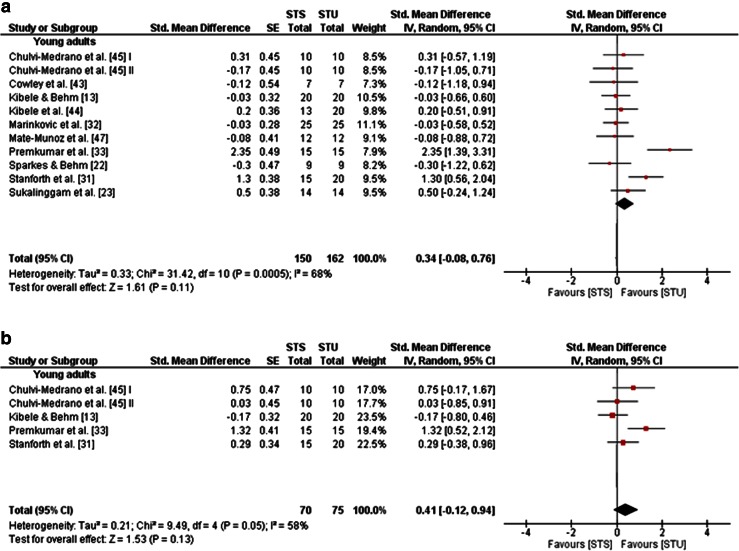
Effects of strength training on unstable surfaces (STU) versus stable surfaces (STS) on measures of maximal strength (**a**) and strength endurance (**b**) in healthy young adults. *CI* confidence interval, *df* degrees of freedom, *IV* inverse variance, *SE* standard error, *Std.* standard

**Fig. 7:**
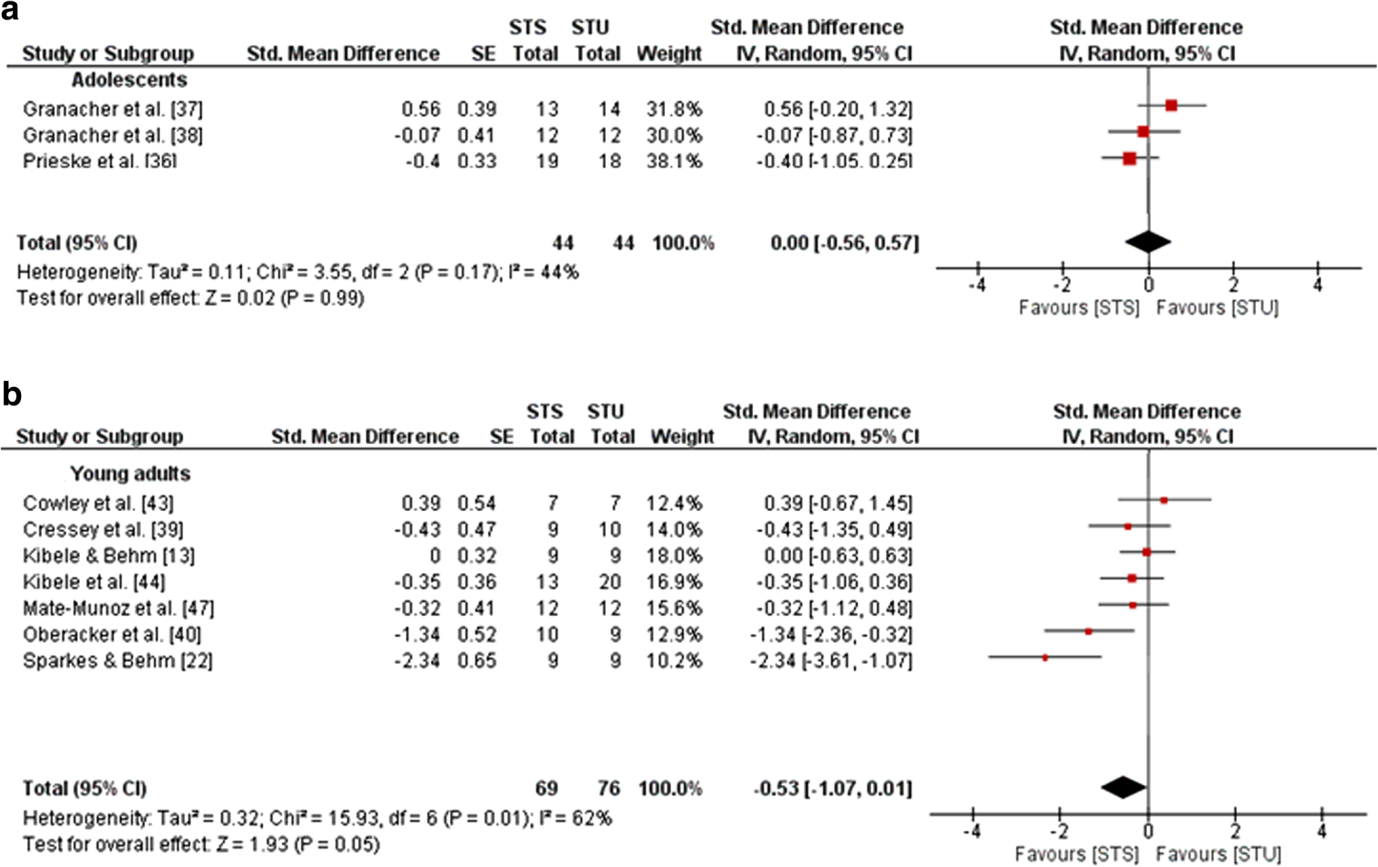
Effects of strength training on unstable surfaces (STU) versus stable surfaces (STS) on measures of muscle power in healthy adolescents (**a**) and young adults (**b**). *CI* confidence interval, *df* degrees of freedom, *IV* inverse variance, *SE* standard error, *Std.* standard

Three studies in adolescents [36–38] and 12 studies in young adults [13, 22, 23, 31–33, 39, 40, 43–45, 47]—but no studies in children, middle-aged and old adults—examined the effects of STU compared with STS on measures of strength, power and balance (Table 1). A general forest plot for measures of muscle strength and balance is illustrated in Fig. 5a, b. Our analyses revealed small effects in favour of STU on muscle strength (mean SMD_b_ = 0.15, *I*^2^ = 68 %, *χ*^2^ = 46.92, *df* = 15, *p* < 0.001; 15 studies [13, 22, 23, 31–33, 36–40, 43–45, 47]; Fig. 5a) and balance (mean SMD_b_ = 0.09, *I*^2^ = 0 %, *χ*^2^ = 0.69, *df* = 4, *p* = 0.95; five studies [13, 22, 37, 38, 44]; Fig. 5b). Additionally, our item-specific analyses revealed inconsistent results as indicated by training-induced changes in favour of STU (i.e. a positive SMD_b_ value), as well as STS (i.e. a negative SMD_b_ value). More specifically, small to medium effects were detected for measures of maximal strength in adolescents (SMD_b_ = −0.66 to −0.28; one study [36]) in favour of STS, and small effects in young adults (mean SMD_b_ = 0.34, *I*^2^ = 68 %, *χ*^2^ = 31.42, *df* = 10, *p* = 0.0005; 10 studies [13, 22, 23, 31–33, 43–45, 47]; Fig. 6a) in favour of STU. In terms of strength endurance, large effects were observed in adolescents in favour of STS and small effects in favour of STU (SMD_b_ = −1.49 to 0.24; one study [37]). In young adults, STU produced small effects on variables of strength endurance (mean SMD_b_ = 0.41, *I*^2^ = 58 %, *χ*^2^ = 9.49, *df* = 4, *p* = 0.05; four studies [13, 31, 33, 45]; Fig. 6b) in comparison with STS. In terms of muscle power, no effects were detected in adolescents (mean SMD_b_ = 0, *I*^2^ = 44 %, *χ*^2^ = 3.55, *df* = 2, *p* = 0.17; three studies [36–38]; Fig. 7a); yet medium effects were observed in young adults in favour of STS (mean SMD_b_ = −0.53, *I*^2^ = 62 %, *χ*^2^ = 15.93, *df* = 6, *p* = 0.01; seven studies [13, 22, 39, 40, 43, 44, 47]; Fig. 7b).

**Fig. 8:**
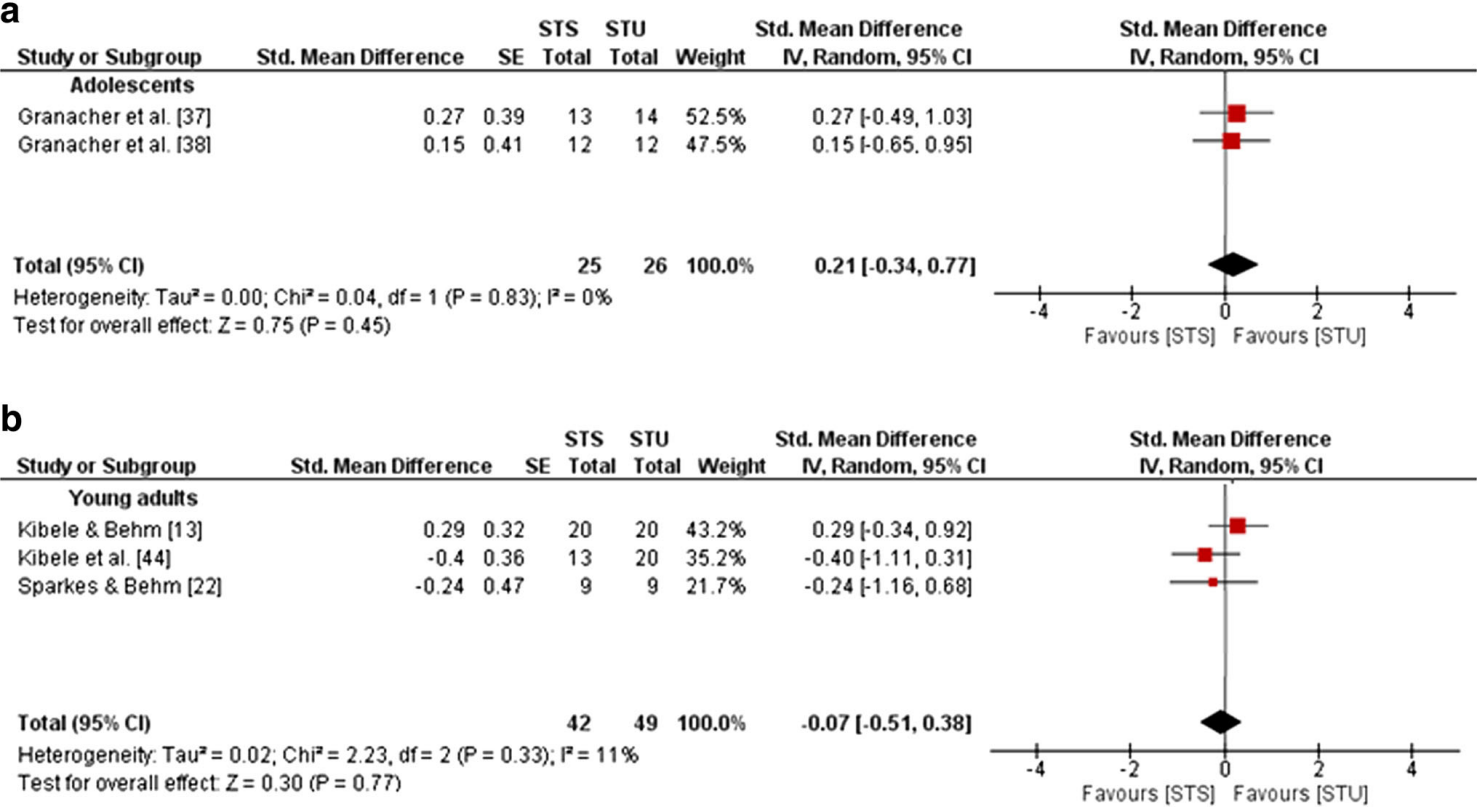
Effects of strength training on unstable surfaces (STU) versus stable surfaces (STS) on measures of static balance in healthy adolescents (**a**) and young adults (**b**). *CI* confidence interval, *df* degrees of freedom, *IV* inverse variance, *SE* standard error, *Std.* standard

**Fig. 9:**
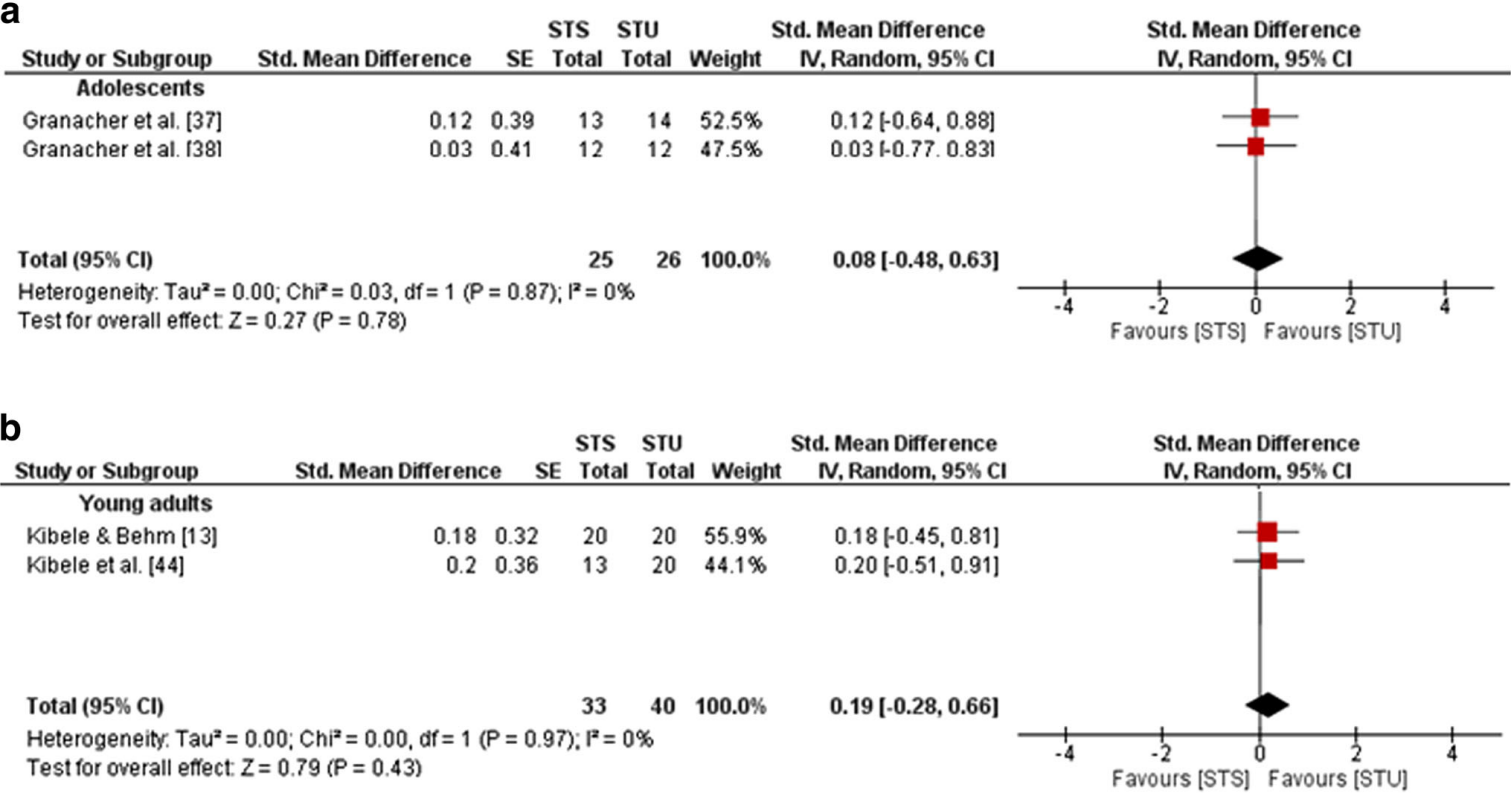
Effects of strength training on unstable surfaces (STU) versus stable surfaces (STS) on measures of dynamic balance in healthy adolescents (**a**) and young adults (**b**). *CI* confidence interval, *df* degrees of freedom, *IV* inverse variance, *SE* standard error, *Std.* standard

Furthermore, training-induced changes in balance performances following STU or STS were obtained, indicating small effects in adolescents for variables of static balance (mean SMD_b_ = 0.21, *I*^2^ = 0 %, *χ*^2^ = 0.04, *df* = 1, *p* = 0.83; two studies [37, 38]; Fig. 8a) and dynamic balance (mean SMD_b_ = 0.08, *I*^2^ = 0 %, *χ*i^2^ = 0.03, *df* = 1, *p* = 0.87; two studies [37, 38]; Fig. 9a) in favour of STU. In young adults, small effects were observed for measures of static balance in favour of STS (mean SMD_b_ = −0.07, *I*^2^ = 11 %, *χ*^2^ = 2.23, *df* = 2, *p* = 0.33; three studies [13, 22, 44]; Fig. 8b) and for measures of dynamic balance in favour of STU (mean SMD_b_ = 0.19, *I*^2^ = 0 %, *χ*^2^ = 0, *df* = 1, *p* = 0.97; two studies [13, 44]; Fig. 9b).

## Discussion

This is the first systematic literature review and meta-analysis to examine the effects of STU on measures of muscle strength, power and balance, administered in the form of controlled trials in healthy individuals across the lifespan. Twenty-two controlled trials (four in adolescents, 15 in young adults and three in old adults) were included in this review. The major findings of this review were that STU as compared with CON is effective in improving variables of muscle strength, power and balance in healthy adolescents, young adults and old adults when tested on stable surfaces. Further, small overall effects were found for measures of strength and balance in adolescents and young adults in favour of STU compared with STS. However, our item-specific analyses (e.g. maximal strength) revealed no consistent advantage of STU when it was compared with STS particularly in adolescents and young adults.

Adherents of STU would propose that such exercises provide advantages over STS, due to training specificity principles (i.e. exercising on unstable devices for unstable task demands during sports, fitness and in the workplace) [3, 4], while the greater degree of instability provides greater stress and thus greater opportunities for training adaptations of the neuromuscular and balance systems [11, 12]. The question of training specificity could not be directly addressed in this review, as the testing measures were typically laboratory based. The studies often used tests such as a one-legged stance, as well as strength and power measures, such as bench press, squat, CMJ, SJ and others, that were performed under relatively stable and stationary conditions (Table 1). The proposed training specificity of STU should be more apparent in tests performed under more unstable conditions (e.g. jumping from or landing on a balance pad). However, a counter-argument would concede that exercises used for STU are also typically static (stationary) exercises (performed without translational movement) and thus STU training specificity would not apply to dynamic, mobile, unstable activities, such as ice hockey, beach volleyball or soccer on a muddy field. For example, static STU [13] and dynamic STU [44] did not provide additional benefit on a dynamic balance test nor on a 20-m hopping test for speed. Balance improvements with the hopping test would have been expected to improve limb power output and decrease contact time, resulting in faster speeds. While Sparkes and Behm [22] found no significant training-specific differences following 8 weeks of static STU and STS, they did report a trend (*p* = 0.06) for the STU group to improve the stable to unstable chest press force ratio to a greater degree (25 %) than the stable group (11 %). Although the trunk is supine upon a stable bench during the bench press, the load is suspended above the body, and the movement must be controlled with appropriate trunk and joint muscle stabilization. However, on the basis of the available data, it seems that STU does not provide systematic training-specific balance advantages over STS, irrespective of the age group considered (i.e. adolescent and young adults).

The lack of superiority of STU for balance measures may illustrate a dose–response or intensity–response relationship. While specific studies comparing the extent of centre of pressure excursions with STU versus STS are not available, it is generally accepted that STS involving free-weight lifts provides moderate levels of instability [1, 8, 9]. The placement and movement of bars, dumbbells and other implements on the shoulders (i.e. squats), above the body (i.e. shoulder presses, cleans, snatches) or in front of the body (i.e. bicep curls), for example, places disruptive torques outside the centre of gravity, challenging the system to maintain equilibrium. Although the challenges to postural stability may be greater during performance of a resistive exercise on an unstable surface, the present results demonstrate that this greater degree of stress does not lead to greater systematic balance improvements in adolescents and young adults.

The lower force and power outputs [1, 8, 9, 20, 49–52]—as well as the decreased movement velocity and range of motion [50]—associated with STU could result in less rigorous strength and power training adaptations. However, not all studies that have investigated the effectiveness of STU reported force reductions under unstable conditions [43, 53, 54]. The Canadian Society for Exercise Physiology position stand [7] warns that “From a performance standpoint, unstable devices should not be utilized when hypertrophy, absolute strength, or power is the primary training goal, because force generation, power output, and movement velocity are impaired and may be insufficient to stimulate the desired adaptations, especially in trained athletes” (p. 110). However, the present results suggest that at least for non-elite athletes, there is a strength and power training stress/intensity plateau that is sufficient to induce positive training adaptations. In their review, Behm and Colado [8] reported that the mean force deficit with STU compared with similar STS exercises was 29 %. The present review indicates that in comparison with STU, exceeding that plateau by introducing greater strength or power challenges with STS does not provide significant advantages across the lifespan. As there were no age-specific differences in the training response, STU can be employed by the non-elite training population to improve strength and power and to achieve functional health benefits. In fact, the approximate STU-induced 30 % force deficit [8] could be viewed alternatively as a benefit, as the lower external forces or torques might decrease the chance or incidence of training-related injuries or might be more beneficial for rehabilitation of an injured muscle group [8].

Furthermore, the American College of Sports Medicine recommends that older adults should conduct strength training using light loads (40–50 % 1RM) at the beginning of training and moderate loads near the end of training (60–70 % 1RM) [55]. Similarly, low- to moderate-intensity strength training is recommended in youth [17]. Hence, the instability-related lower force outputs during performance of STU may not represent a compromising issue regarding neuromuscular adaptive processes in old adults and youth. Further, there is evidence in the literature that even in young healthy adults, strength training using low compared with high loads is equally effective in enhancing muscle strength. For example, isometric strength training at 100 versus 60 % of maximal voluntary contraction (MVC) [56] or dynamic strength training at 55–60 versus 80–90 % 1RM [57] resulted in similar improvements in measures of muscle strength. Thus, there is evidence that application of lower loads during STU provides a sufficient training stimulus to ensure similar strength or power training gains in comparison with STS using higher loads in different age groups. Of note, the study methodology of the included studies has to be taken into account when interpreting our findings. In other words, the included training studies ranged from 2 to 12 weeks and were conducted in primarily untrained or recreationally active individuals.

A potential reason for the observed similar training-induced adaptations following STU compared with STS could be related to similar or even higher levels of muscle activation during performance of STU [49, 58]. In fact, Anderson and Behm [49] found no significant differences in overall electromyographic (EMG) activity of trunk and shoulder muscles during performance of a chest press exercise on an unstable surface (i.e. a Swiss ball) as compared with a stable surface (i.e. a bench). Further, significantly higher trunk muscle activity was observed during performance of squat movements on an unstable surface (i.e. performed with a balance disc under each foot) versus a stable surface (i.e. performed with a Smith machine) [58].

Our findings did not support our initial hypothesis regarding the greater effectiveness of STU in youth compared with adults. When comparing STU with CON, our results revealed medium effects of STU on measures of maximal strength in young adults and, depending on the analysed parameter, no effects (i.e. maximal strength of the trunk rotators right) to medium effects [i.e. maximal strength of the trunk flexors, rotators, lateral flexors (left/right), lateral rotators (left)] in old adults. On the basis of these results, it can be concluded that STU provides an adequate stimulus to increase maximal strength in seniors, which is mostly equal to that observed in young adults. Further, comparison of STU and STS revealed similar training-induced performance enhancements for muscle endurance and dynamic balance in both adolescents and young adults. Methodological reasons may account for this somewhat unexpected finding. However, in terms of training, similar core strength, as well as plyometric exercises, on unstable surfaces/devices were included in adolescent [36–38] and adult [23, 31] training protocols. Further, in terms of testing, differences in the sensitivity of the applied strength, power and balance tests may also have been responsible for the unexpected findings. Yet, similar test modalities (e.g. CMJ, one-legged stance), equipment (e.g. force plate) and parameters (e.g. jump height, postural sway) were used in studies that investigated adolescents [36, 38] and young adults [44, 47]. Therefore, methodological reasons appear not to be responsible for our findings. This is why we suggest that instability-related reductions in absolute training loads during STU as compared with STS may explain our results because the reduced loads are not challenging enough to induce extra adaptive processes in the adolescent neuromuscular system. In fact, adolescence is characterized by significant increases in levels of circulating androgens (e.g. testosterone), particularly in boys [59, 60], which is why high training loads appear to be more suitable to induce marked increases in muscle mass and strength in this age group. As a limitation of this study, it has to be noted that only four studies were found that investigated the impact of STU versus STS or CON in adolescents and only three studies were found that investigated the impact of STU versus STS or CON in old adults. On the basis of the rather small number of studies, we consider our findings as preliminary. Therefore, further research is needed to determine the general effectiveness of STU as compared with no training or regular training. Equally or even more important is the need to elucidate the specific effects of STU as compared with other strength training programs (e.g. STS using high/low loads).

## Conclusions

This systematic review and meta-analysis shows that STU, when compared with no training or regular training only, is effective in improving strength performance in adolescents, young adults and old adults, as well as power and balance performance in young and old adults. However, heterogeneous effects were particularly found in adolescents and young adults when the effects of STU were compared with those of STS. Therefore, we conclude that the application of STU compared with STS has limited additional effects on measures of muscle strength, power and balance in healthy adolescents and young adults. Therefore, the use of unstable as compared with stable surfaces during strength training is only partially recommended. Because our systematic literature search did not identify studies that investigated the effects of STU versus STS in children, middle-aged adults and old adults, further research of high methodological quality (i.e. randomized controlled trials) is needed to determine whether there are extra effects of STU on muscle strength, power and balance performances in those age groups.
